# Long axial field of view PET scanners: a road map to implementation and new possibilities

**DOI:** 10.1007/s00259-021-05461-6

**Published:** 2021-06-16

**Authors:** Riemer H. J. A. Slart, Charalampos Tsoumpas, Andor W. J. M. Glaudemans, Walter Noordzij, Antoon T. M. Willemsen, Ronald J. H. Borra, Rudi A. J. O. Dierckx, Adriaan A. Lammertsma

**Affiliations:** 1 Medical Imaging Center, Department of Nuclear Medicine and Molecular, University of Groningen, University Medical Center Groningen, Hanzeplein 1, PO 9700 RB, Groningen, The Netherlands; 2 Department of Biomedical Photonic Imaging, Faculty of Science and Technology, University of Twente, Enschede, The Netherlands; 3 Leeds Institute of Cardiovascular and Metabolic Medicine, School of Medicine, University of Leeds, Leeds, UK

**Keywords:** PET, Field of view, Diagnosis, Implementation, Financial, Opportunities

## Abstract

In this contribution, several opportunities and challenges for long axial field of view (LAFOV) PET are described. It is an anthology in which the main issues have been highlighted. A consolidated overview of the camera system implementation, business and financial plan, opportunities and challenges is provided. What the nuclear medicine and molecular imaging community can expect from these new PET/CT scanners is the delivery of more comprehensive information to the clinicians for advancing diagnosis, therapy evaluation and clinical research.

## Introduction

After its commercial introduction in 1978 [1], positron emission tomography (PET) was primarily used as an advanced in vivo imaging technique to measure regional physiology non-invasively, with an initial focus on pathophysiological studies of the brain [2]. One of the first widespread clinical applications was the assessment of myocardial viability [3]. It was not until the mid-1990s, however, before PET really entered the clinical domain [4]. The development of whole-body PET acquisitions made it possible to survey the entire body [5]. Using this technique, it was subsequently shown that [^18^F]FDG (whole-body) PET was the most sensitive method for detecting metastases, which led to its acceptance as a landmark clinical diagnostic tool in oncology [6, 7].

Using a conventional PET scanner, a whole-body scan is acquired by moving the patient bed through the gantry. This is needed because of the limited axial field of view (FOV) of 15 to 25 cm of conventional scanners. Although sensitivity has steadily increased over the years and, more recently, with the introduction of digital PET systems [8], at present, the typical duration of a conventional whole-body PET/CT scan, as available in the majority of imaging institutes, is still relatively long (i.e. 10–30 min) in order to acquire sufficient counts for the entire body. A limitation of whole-body imaging (essentially a series of successive static scans) is that it renders the acquisition of dynamic scans less practical and, consequently in a clinical setting, images usually are analysed qualitatively (e.g. visually) or semi-quantitatively (e.g. standardised uptake value (SUV) or tumour-to-blood ratios (TBR)) [9].

For [^18^F]FDG, the most common tracer used in clinical practice, semi-quantitative SUV analysis is an acceptable approach, as there is generally a good correlation between SUV and glucose metabolism derived from dynamic PET scans. When used for response monitoring purposes, however, one should be aware of a possible dissociation between these parameters, e.g. due to a change in plasma clearance that is not accounted for in the SUV calculation [10]. For most other tracers, however, the relationship between SUV and the underlying biological parameter of interest is less clear. This is due to the fact that SUV does not only represent the biological parameter of interest, but also contains free and non-specific signals. These contributions to the overall (SUV) signal can be so large that any differences in the specific biological signal may be lost. A clear example is given by [^11^C]erlotinib, where both SUV and TBR, derived from static scans, do not show significant differences between tumours with and tumours without mutated epidermal growth factor receptors (EGFR), in contrast to the volume of distribution (*V*_*T*_) derived from dynamic scans [11].

Although PET has the potential to become an important non-invasive (whole-body) tool in precision medicine, the [^11^C]erlotinib example illustrates a current dilemma. In principle, PET could be used to predict response to therapy, as higher [^11^C]erlotinib uptake in lesions indicates better response to erlotinib therapy. For a single lesion, this could be achieved using *V*_*T*_ derived from a dynamic scan. Although, in theory, it is possible to perform a dynamic scan by acquiring multiple single bed acquisitions repeatedly or, more recently, using a continuously moving bed acquisition protocol, the temporal resolution of such an approach is only sufficient for some selected applications with slow kinetics (such as Patlak analysis of [^18^F]DG uptake). In the majority of cases, however, kinetic analysis requires higher temporal resolution that can only be achieved by dynamic scanning of a predefined single bed position. As overall response will depend on the “poorest” lesion, it is possible that this lesion will be missed. To assess all lesions, a whole-body scan would be required, but this means the acquisition of static scans for successive bed positions and, at least for [^11^C]erlotinib, such scans are non-informative [12].

The [^11^C]erlotinib example is not unique. It applies to most tracers where the (specific) signal of interest is only part of the total PET signal. The dilemma whether to perform a (static) whole-body scan, covering all lesions, or an accurate dynamic scan over a more limited axial FOV can be resolved with the newest generation of PET/CT scanners, a so-called long axial field of view (LAFOV) PET [13]. Although the term total-body PET has been used for such a scanner, strictly speaking, this is only correct for systems with an axial FOV of at least 2 m. To be more general, the term LAFOV PET seems more appropriate, indicating any scanner with a significantly extended axial FOV (longer than 1 m), enabling dynamic acquisitions of all (body) lesions simultaneously. In addition, according to NASA’s anthropometric dimensional data, the torso and head of 95% of the USA male population will be within the axial field of view of a 1-m-long PET scanner, therefore providing simultaneous coverage of all major internal organs [14].

An important additional advantage of a LAFOV PET is the substantial increase in sensitivity (a factor of 10 to 40 depending on the volume of detector material, i.e. the actual length of the scanner) compared with a conventional PET scanner [15]. This, in turn, allows for faster scans (e.g. higher patient throughput) and/or an appreciable reduction in injected dose [16], leading to new clinical indications, patient populations, and research possibilities [17]. This article describes the opportunities LAFOV PET systems offer, together with what is needed for installation in a university medical centre (i.e. clinical support, business plan and change of infrastructure) and new challenges that need to be addressed in order to make optimal use of this new system.

## New clinical and research applications

### High patient throughput

As mentioned above, the higher sensitivity provides an opportunity for faster acquisitions and thus allows for scanning more patients within the same time frame. In terms of patient throughput, it may be possible to scan as many as 6–8 patients per hour, provided that patient setup can be streamlined. As tracer production corresponds to a substantial part of overall costs, this could lead to an important reduction in scanning costs (i.e. more patients can be scanned with the same tracer batch). Furthermore, a reduction in acquisition time may make it easier for patients who are less mobile (e.g. intensive care patients), who normally need sedation (e.g. claustrophobic or extremely nervous patients and children) or who are in a lot of pain (e.g. oncology patients).

### High-risk population screening

Alternatively, the higher sensitivity may be used to lower the injected dose, which in turn will result in a lower radiation burden for the patient. It may even be possible to reduce the injected dose to such an extent that LAFOV PET/CT could be used for screening. This may be very relevant for high-risk populations of any disease in which early detection may substantially increase the potential of successful treatment. For example, conventional PET/CT when combined with blood testing has emerged as a potential screening tool for early cancer diagnosis and guided interventions [18]. Likewise, targeted screening for prospective patients with dementia, where pathological amyloid depositions in the brain may accumulate 10 to 20 years before manifestation of the first symptoms [19] or cardiovascular diseases may lead to increased treatment efficacy and a prolonged high quality of life.

### Paediatric imaging

The possibility of reducing radiation dose is also helpful when scanning children, whose cells are more sensitive to radiation damage. Paediatric patients are frequently diagnosed with malignant diseases that are characterised by a diffuse pattern of involvement. For example, haematological malignancies, such as lymphomas and leukaemia, which involve both bone marrow and associated organs (e.g., lymph nodes and spleen) [20], account for a large proportion of paediatric cancers. Apart from reducing injected dose, the faster scanning option of a LAFOV PET/CT may also be useful in children, as it can help reduce motion artefacts and it may limit the need for anaesthesia, which is a substantial additional obstacle for performing repeated PET/CT scans during a course of treatment.

### Drug development

As mentioned earlier, PET has potential as a non-invasive tool in precision medicine. Similarly, PET may facilitate and improve efficacy of the drug development process, a process that is known to be highly inefficient and expensive [21]. At present, however, a major limitation is the maximum radiation dose permissible, which often limits the number of scans that can be performed during a trial, especially in volunteers. The lower doses that are possible with a LAFOV PET/CT scanner also means that there are less restrictions in the number of scans, which provides more flexibility (e.g. combination of different tracers to look at different biological aspects) and a role of PET in more accurately measuring full-body pharmacodynamics.

### Guided therapy

Many (particularly oncology) patients undergo PET/CT scans on a regular basis, especially within the context of response evaluation (e.g. every three cycles in case of immune therapy). Reduction in administered dose offers the possibility of more frequent treatment assessments, and consequently the possibility to identify non-responders at an earlier stage, thereby offering the opportunity to terminate non-effective treatment earlier, and potentially switch swiftly to another, more successful treatment. The lower dose also enables repeat scans using labelled monoclonal antibodies that are associated with a higher radiation dose due to the need for a longer lived radionuclide such as ^89^Zr and ^90^Y. In addition, the improved sensitivity will result in improved image quality of radionuclides with these low positron abundance radionuclides. Ïn addition, the lower administered dose allows for a combination of multiple tracers in the same patient (molecular fingerprinting).

Furthermore, the improved sensitivity profile of the LAFOV PET scanner enables acquisitions at later time points, i.e. up to five additional half-lives of a radiotracer. This may be important for several applications, e.g. for antibody imaging (which are characterised by very slow kinetics) [21].

Finally, the extended field of view will be convenient for assessing the biodistribution of novel radiotracers, as multiple organ axes are captured in a single scan and in combination with the dynamic acquisition kinetic information can prove valuable for more precise prediction of therapeutic efficacy, as illustrated by the erlotinib example.

### Organ axes

It has become increasingly apparent that many diseases and conditions, originally thought to be confined within a single organ, are much more complex and involve the interplay of that organ with other organs or systems [22]. As an example, the gut–brain axis is a feature in neuroscience. Bacteria in the gut could have profound effects on the brain, and might be tied to a whole family of disorders [23]. There is also evidence that gut microbiota and their metabolites interfere with the host’s immune and endocrine systems [24]. Here, LAFOV PET scanners could be helpful by dynamic imaging of the immune system to track and trace immune cells of the gut that interact with adjacent as well as distant organs and tissues.

The work of Per Borghammer and colleagues advocates a retrograde involvement of the brain in idiopathic Parkinson’s disease. A caudorostral gradient of dysfunction as shown by molecular imaging supports the hypothesis that a-synuclein pathology in Parkinson’s disease initially targets peripheral autonomic nerves and then spreads rostrally to the brainstem [22, 25, 26]. Imaging studies that enable combined assessment of brain and spinal cord will provide information on the molecular basis of neurodegenerative diseases that originate in the brain, but extend into the spinal cord and potentially lead to partial or complete paralysis. The spinal cord is involved in several neurological disorders and a more detailed assessment of its relationship with the brain may be useful not only in personalising treatment, but also in identifying treatment targets and quantifying disease progression. Examples include elucidation of the role of microglial activation in motor neuron disease using, for example, [^18^F]DPA-714 [27] and the pathophysiology of progression in multiple-sclerosis, hereditary spastic paraplegia and unexplained medical myelopathy.

There is also evidence that connects cardiovascular function, neurochemical asymmetries and depression [28]. The brain–heart axis is further implicated in post-stroke cardiovascular complications known as the stroke-heart syndrome, sudden cardiac death and the Takotsubo syndrome, amongst other neurocardiogenic syndromes. In the early 1990s, a dynamic [^15^O]H_2_O PET brain study identified the central nervous pathways of angina pectoris, highlighting the interplay between the brain and the heart in such patients [29]. One other obvious example would be the emerging evidence of the role of acute myocardial infarction (MI) that triggers both local and systemic inflammatory responses. PET imaging with [^11^C]methionine specifically identifies an astrocyte component, enabling further dissection of the heart-brain axis in post-MI inflammation [30]. Another recent [^18^F]FDG PET/CT study has linked resting amygdalar activity with cardiovascular events, indicating a potential mechanism to predict risk of cardiovascular disease caused by stress [31]. Within the last decade, novel targeted therapies for multiple pathophysiological mechanisms have been identified [32].

Appropriate interpretation of changes in markers of kidney function is essential during treatment of both acute and chronic heart failure. Better understanding of the role of cardio-renal interactions in heart failure is essential with respect to symptom development, disease progression and prognosis. Using LAFOV PET scanners, organ interactions can be studied before and during therapy. Again, a better understanding of these interactions may lead to precision medicine for individual patients [33]. What is unique about the LAFOV PET scanners is the inclusion of all relevant organs in a single FOV, enabling novel approaches to study physiological or pathophysiological interactions between organs, including brain–heart-kidney-liver-gut interactions.

### Infection and inflammation

Infection and inflammation imaging has significantly expanded over the years, with special focus on gathering evidence-based data for clinical indications and providing guidelines and diagnostic flowcharts in collaboration with clinical societies. The arrival of the LAFOV PET/CT systems will provide a further boost to this field. Using current SAFOV PET/CT scanners, false-negative scans can be expected in patients with low-grade chronic infections, bacterial growth on biofilms at the joint prosthesis or vascular graft site, in small infectious foci, or in infections with a small number of bacterial colonies [34, 35]. The LAFOV PET/CT scanner may reduce this problem and may lead to an increase in diagnostic accuracy in patients with bacteraemia, endocarditis, vascular graft infections and fracture-related infections.

As mentioned earlier, the increased sensitivity of LAFOV PET makes it possible to reduce the injected dose. This is especially important for the field of infection and inflammation, as it opens up the possibility to use labelled monoclonal antibodies (so-called immunoPET) also for this indication. As immunoPET is based on radionuclides with a longer half-life (such as ^89^Zr), at present, it is used almost exclusively in oncological patients with a relatively short life expectancy. By reducing the injected dose, immunoPET will also become feasible in younger patients with inflammatory diseases.

Another important aspect is a possible shift from invasive procedures to non-invasive molecular imaging. An example is the follow-up of children and young adults with an inflammatory bowel disease (Crohn’s disease or ulcerative colitis). Nowadays, follow-up is performed yearly using an invasive colonoscopy under sedation. With LAFOV PET, this may be performed non-invasively within a few minutes using either [^18^F]FDG or (in the future) more specific tracers without the need for sedation.

### A potential game changer in research and clinic

The intrinsic strength of PET imaging is its ability to track the position of single annihilation events with infinitesimal precise timing information (i.e., fraction of a nanosecond [36]. This means that, in practice, the (imaging) temporal resolution is limited only by the sensitivity of the scanner. A substantial increase in counted annihilation photons will make it possible to improve statistics and reconstruct much shorter frames with acceptable signal to noise ratio (SNR). For example, the feasibility of 100 ms duration frames was recently shown as part of a 3D animation produced by a team at University of California, Davis [37]. This sensitivity boost allows to literally follow the radiotracer molecules whilst they travel within the human body and, due to the extended FOV of the scanner, measure kinetics for a range of organs [38]. More than that, it has recently been shown that fast kinetic parameters such as *K*_*1*_ can be derived from only a couple of minutes of an early dynamic scan [39]. Dynamic PET becomes more practical, straightforward, simpler and more powerful than ever before, and with the use of appropriate kinetic models it will provide more accurate, precise and reliable quantification about the body ecosystem, which will be a game changer in both clinical practice and research [40].

Another key advantage is dose reduction. Beyond the obvious, but important benefit of imaging more radiosensitive populations such as children and pregnant women at lower dose [41], imaging will enable wider use of ^18^F radionuclides for scanning volunteers, potentially more than once, without the need for dosimetry approval [42], something which will definitely be the case for ^11^C-labelled tracers [43]. The improved access to people without any underlying conditions will create a fast-track route for protocol optimisation and clinical translation of new or even existing radiotracers within a wider range of clinical conditions.

Finally, as already mentioned, lower administered doses provide the possibility to perform PET scans with multiple tracers in the same patient [44]. As such, LAFOV PET offers exciting opportunities to image a wide variety of targeted functions at the same point in time, thereby providing more detailed insights into the biology of diseases at a whole-body level.

## Clinical and board support

The purchase of any camera, and certainly of such an expensive PET/CT system, requires approval by the hospital management. This was obtained by generating the usual business case and highlighting the benefits of LAFOV PET over standard systems, thereby focusing on the new possibilities for both the clinic and medical research. Somewhat surprisingly, the application was supported by all hospital departments, not only those who traditionally are involved in PET (e.g. neurology, cardiology and oncology), but also from those who are not familiar with PET. This clearly illustrates that LAFOV PET already has raised interest amongst disciplines that have not been active in molecular imaging yet. Clearly, this interest (and concrete plans) has been a strong argument in obtaining approval from the hospital management.

## Business/financial plan

Given the advantages, one question behind the installation and further widespread adaptation of LAFOV PET is its high costs that makes it unaffordable for several hospitals even in high-income countries [45]. Thus, a realistic business plan could focus on the potential of a LAFOV PET/CT scanner for increased patient throughput. However, in order to achieve this increase, several prerequisites need to be met, including (1) allowing for only a moderate decrease in injected dose compared with that used on conventional PET/CT scanners; (2) radiochemistry logistics being able to keep up with rapid successive injections; (3) patient facilities, such as preparation room, waiting room and changing rooms being available for a higher number of patients; and (4) additional personnel being available to aid with increased patient logistics. Interestingly, clinical practice in radiology and nuclear medicine has shown that introduction of higher-sensitivity systems (e.g. digital PET replacing analogue PET, 3 T MRI replacing 1.5 T MRI) usually is invested more in better image quality than in faster scans [8, 46]. These considerations should be included in the business plan to avoid overly optimistic expectations on the number of patients who can be scanned per day using a LAFOV PET/CT.

However, if these new opportunities are materialised in clinical practice, a substantial increase in the use of PET as a diagnostic and therapeutic guidance tool can be anticipated. Although, at present, it may be financially difficult for some centres to replace their conventional scanners with a longer one, it may be the natural direction once there is a demand for increased scanning capacity. Once this happens, it will be cheaper for one centre to increase its scanning capacity by replacing a conventional PET/CT scanner with an extended FOV PET/CT scanner rather than by two or three conventional PET/CT scanners, as the first option will need less space and less refurbishment.

A longer-term business plan should consider a substantial expansion of PET usage in other patient populations (e.g. paediatric) as well as screening healthy individuals with higher risk of specific diseases. Furthermore, the advantages offered by dynamic PET acquisitions for quantification (i.e. essentially increasing specificity and accuracy of acquired signals) would make LAFOV PET an essential and commonly used tool for more comprehensive guidance of many additional therapies.

## Infrastructure

General information regarding the required infrastructure for any PET/CT scanner or even a clinical PET centre is widely available, e.g. by the International Atomic Energy Agency (IAEA) Human Health Series [47]. A clinical PET centre should have several rooms including, but not limited to, a general reception area with waiting area, a preparation room for tracer administration, a “hot” uptake room where patients stay for a certain period of time after injection to allow for sufficient uptake in the body (e.g. 60 min for [^18^F]FDG) and of course the PET/CT scanning room(s). In addition, a separate control room is required as well as rooms for auxiliary equipment and staff. A dedicated toilet for patients is required to prevent radioactive contamination of staff and visitors/personnel. Depending on the scope of the centre, a cyclotron and good manufacturing practice (GMP) laboratories for production and dispensing of activity may be required or, if activity is purchased, a simple hot-cell for storage and unpacking may be sufficient. The remainder of this section will focus on special considerations when installing a LAFOV PET/CT scanner, in particular the scanner room and its shielding, the IT infrastructure and the operating requirements.

Structural modifications of the scanner room and floor depend on the actual system to be installed, with LAFOV PET/CT potentially requiring modifications of rooms initially optimised for traditional PET/CT scanners. An important advantage of LAFOV scanners with intermediate length such as the Siemens Biograph Quadra (106-cm-long FOV) is that, in principle, they can be installed within a room that was previously used for a traditional PET/CT scanner because of the similarity in footprint. However, care should be taken to ensure that the additional weight of the device does not exceed the structural limits of the floor and that the extended FOV still allows for optimal logistics in the space around the scanner. Placing the device at a slight angle within the room can create additional space and allows access for maintenance.

The amount of shielding depends on the size of the room, the workload of the system and local regulations. Typically, 2–3-mm lead is required from floor to ceiling to shield for CT radiation. This includes windows which need to be from lead glass. To shield for the 511 keV annihilation radiation, higher amounts of shielding may be required, which can easily exceed 10 mm of lead. If the extended PET/CT is used solely in combination with considerable lower injected doses than for traditional PET/CT systems, less shielding may be appropriate, thereby reducing the costs for the scanner room. However, this would significantly limit the range of applications and would especially prevent dynamic and delayed imaging studies where higher amounts of radioactivity are still needed.

As mentioned above, one of the key features of LAFOV PET/CT is the ability to obtain complete dynamic PET data, which can be reconstructed offline into datasets with high temporal resolution. However, this requires storage of the raw (list mode) data, which can quickly amount up to several terabytes per hour of scan time, depending on the characteristics of the tracer used and the injected dose (as the amount of recorded data is proportional to the number of counts). Nevertheless, for scientific use and artificial intelligence (AI) applications, storage of these list mode data is essential. This vast amount of data poses significant challenges for almost any existing hospital infrastructure at present. Although centralised storage of data at hospital IT facilities would be preferred, this might not always be possible, because a dedicated communication architecture (several 10 Gbps network lines) would be required to centralised storage media. Therefore, possibly an acceptable and cost-effective option that does not disrupt existing hospital IT systems is to implement a storage buffer in the equipment room (e.g. 1 Petabyte RAID array) that can rapidly transfer recently acquired data back and forth to the PET/CT reconstruction workstation, and additionally move older data overnight to centralised hospital storage facilities.

All scanners come with a site planning guide, which stipulate the conditions under which they should be used. In particular, cooling of the scanner requires attention, as digital PET detectors require a constant temperature in order to function reliably. Considering the large number of detectors in the extended FOV, these scanners can require several times the amount of cooling that is needed for traditional scanners, which may require upgrades of the hospital cooling system.

For kinetic modelling and quantification, a system to invasively measure radiotracer concentrations in arterial blood over time will be essential, at least until robust non-invasive solutions for calculating the arterial input function have been developed. Depending on the length of the scanner, easy access to the radial artery may be limited. Online blood withdrawal systems should be carefully evaluated, especially since longer lines between artery and detector result in more dispersion of the measured blood curve.

Standard respiratory and cardiac gating systems as well as optical trackers for real-time motion detection that are compatible with longer scanners would be important additional tools for enabling quantification. Simple restraint systems for minimising bulk motion of the body extremities are also advisable.

## Validation

Upon installation of any PET system, validation according to the National Electrical Manufacturers Association (NEMA) protocols should be performed to ensure reproducibility with other PET systems. Spatial resolution, scatter fraction, noise-equivalent count rate, sensitivity and image quality should be performed according to NEMA NU 2–2012, whereas NEMA NU 2–2018 includes protocols for co-registration accuracy and time-of-flight resolution [48, 49].

NEMA determines spatial resolution as the full width at half maximum of the point-spread function, using a point source at several positions in the FOV, and reconstructing the data without attenuation and scatter corrections. Both NEMA protocols mentioned above contain details on the recommended point source, with the 2018 protocol stressing that systems with smaller crystal size will benefit from using a 74 kBq ^22^Na over the 3.7 kBq [^18^F]FDG point source specified in the 2012 protocol.

For scatter correction and subsequent determination of the noise-equivalent count rate, a phantom consisting of a 70-cm-long polyethylene cylinder, with a line source inserted, is used to acquire data for 12 h. A total of five 70-cm-long polyethylene tubes with an inner diameter of 1 mm are used to eventually determine the system’s sensitivity. Image quality is evaluated using the PET NEMA NU2 image quality phantom positioned in the centre of the FOV. This phantom consists of six spheres, of which four are filled with radioactivity (spheres to background ratio = 8:1), and two are filled with non-radioactive water. These phantoms may need to be extended sufficiently for a more complete performance evaluation of LAFOV systems and formal adaptations of NEMA protocols will be helpful.

Co-registration accuracy is determined by calculating the 3D vector between CT and PET centroids, which are determined by measuring a vial containing radioactivity and contrast agent at six different positions. The NEMA NU 2–2018 protocol describes a new method to calculate time-of-flight resolution from scatter data used for the noise-equivalent count rate [50]. Finally, to comply with the new European Association of Nuclear Medicine Research Ltd. (EARL) guidelines, it is recommended to measure the system’s EARL performance according to standardised reconstructions.

A separate characterisation that needs to be considered is the background intrinsic radiation emitted by the ^176^Lutetium-based scintillators. Due to the large amount of crystal material, this background radiation may pose a limit for low-dose scanning protocols. If substantial, it needs to be accounted for in image reconstructions as part of the background correction.

## Challenges

To fully exploit the exciting opportunities of LAFOV PET/CT scanners, several practical challenges need to be tackled. First, it is likely that the extent of the axial FOV will make it more difficult to scan patients with claustrophobia (despite the shorter acquisition time) and, as mentioned above, withdrawing arterial and venous blood samples may also be more difficult, as both radial artery and antecubital vein will be less accessible. In addition, as already mentioned, storing and processing of the enormous datasets are a challenge for the IT infrastructure. Nevertheless, it is expected that these practical issues will be resolved in the near future.

Next to the practical issues, there are also some more basic challenges, especially when capitalising on the full power of LAFOV PET systems for deriving reliable kinetic information. The first challenge is related to any type of body movement, including that of body extremities, as well as respiration and cardiac contraction. Due to the fully 3D nature of the PET acquisition, motion artefacts may be more pronounced in longer scanners than in conventional scanners. Systematic research efforts have not managed to sufficiently resolve this challenge in conventional PET scanning and it remains a key bottleneck for quantification of dynamic (body) studies. However, the high SNR of LAFOV PET systems provides some confidence of mitigating this issue successfully [51].

The second challenge is related to the invasive procedure of measuring the arterial input function, which is an essential step in extracting kinetic information. The high SNR of LAFOV PET scanners, however, provides an opportunity to produce non-invasively an image-derived input function (IDIF), as in most cases both the heart and the aorta will be in the FOV. In addition, an important advantage of an IDIF is that it is not prone to dispersion, which has to be taken into account when measuring arterial concentrations using an (external) online sampling system. Moreover, it will even be possible to correct for differences in arrival times at the different organs (since apart from those organs, also their feeding arteries are within the FOV). Indeed, impressive dynamic data from the nearly 2-m-long PET scanner at the University of California, Davis, have indicated that the arterial input function could be measured with sufficiently high rates immediately after radiotracer injection, thus providing a new potential for translating kinetic analysis in clinical practice [37]. For some tracers, such as [^18^F]FDG and [^15^O]H_2_O, arterial whole blood concentrations are sufficient for determining the arterial input function. For most other tracers, however, the metabolite-corrected arterial plasma curve is needed as input function, which means that arterial samples are still required to determine the fraction of parent compound. The availability of many organs in the field of view (with different kinetics) provides a means to develop more comprehensive modelling methods (e.g. simultaneous fitting) that will encompass formation of labelled metabolites non-invasively [52] thereby obviating the need for arterial sampling altogether.

LAFOV PET provides a possibility to obtain kinetic data for all organs within the FOV, thereby making it possible to quantify the underlying molecular targets and interactions. The third challenge for LAFOV PET is that it requires kinetic analyses of huge datasets (especially if performed at the voxel level) combined with the fact that the optimal pharmacokinetic model may be different for different organs. Furthermore, the fact that the sensitivity of the scanner may vary substantially along the FOV requires careful consideration. To handle these complexities in routine clinical practice or even research, some form of automation seems to be mandatory. This may be achieved by using AI and organ segmentation in combination with data-driven methods such as cluster and spectral analysis, ideally generating parametric images, i.e. quantitative images of a molecular parameter of interest rather than simple (and inaccurate) uptake images. Clearly, routine use of this option will also require a radical change in mentality in future clinical practice, i.e. favouring longer but more accurate scans over shorter but cheaper uptake scans. This can only be achieved by careful research studies documenting the additional value of fully quantitative scans, keeping in mind that in this era of precision medicine patients deserve the best approach.

## Conclusion

In this contribution, several opportunities and challenges for LAFOV PET have been described. Clearly, this is an anthology in which the main issues have been highlighted. A consolidated overview of opportunities and challenges is provided in Table 1. What the nuclear medicine and molecular imaging community can expect from these new PET/CT scanners is the delivery of more comprehensive information to the clinicians for advancing diagnosis, therapy evaluation and clinical research.

**Table 1:** Opportunities and challenges of long axial field of view PET scanners

New opportunities	Challenges
Improved image quality and high signal to noise	
• Decrease population sampling size which will lead to faster translation of new/existing radiotracers • No need for post-filtering • Reconstruct images at smaller voxel size • Measure/correct motion using PET data • Increase specificity/sensitivity • Increase reproducibility • Detect low-grade uptake • Detect disease at earlier stages	• Amend standardisation values/harmonisation • Substantially non-uniform sensitivity along the axial field of view
Short imaging protocols	
• High patient throughput • Avoid anaesthesia in children • Scan ICU patients • Reduce motion artefacts • Reduce patient inconvenience and consequently increase compliance • Increase screening high-risk but otherwise healthy individuals	• More hot uptake rooms needed
Imaging with lower doses (as many more otherwise lost counts can be measured)	
• Imaging radiosensitive populations (paediatrics, pregnant women) • Imaging larger populations which will lead to faster translation of new/existing radiotracers and better understanding of health/disease • Less affected by radiophobia, which will help replace other diagnostic procedures • Use radiotracers with poor labelling efficiency or low positron emission branching ratio • Wider acceptance for use in clinical trials • Imaging the same patient more sessions, which will lead to frequent/accurate therapy evaluation	• Still requires CT for attenuation correction, which poses a significant limit to dose reduction (unless CT scan is avoided.) • Intrinsic ^176^Lutetium radiation adds background noise
Imaging kinetics with greater temporal range	
• Imaging slower biological processes (e.g. radionuclide therapy and immunotherapy) • Imaging faster biological process due to higher temporal sampling • Imaging biological processes for much longer half-lives of the radiotracer • Derive accurate input function from short frames • Extract more relevant/valuable information from one scan • Increase specificity/sensitivity • Increase reproducibility • Identify input function delay in different organs • Enabling imaging more than one tracer simultaneously	• Require accurate motion correction • Require input function • Require metabolites • Require appropriate kinetic model due to kinetic heterogeneity • Slow computational times for reconstruction, data corrections and kinetic modelling
Imaging longer axial FOV	
• Imaging multiple regions at the body and investigate potential correlations • Biodistribution of newly developed (potentially radiolabelled) drugs • Improve accuracy of dose estimation for radionuclide or other radiomolecular therapies • Measure input function from aortas	• Requires checks of structural limits of the floor • Expensive (when compared to conventional PET) • May require bigger space • Makes difficult to withdraw blood samples or other interventions during the scan • Claustrophobic patients will be harder to scan • Increases environmental footprint • Adaptation of current QA procedures with longer phantoms which can be more difficult and time-consuming to handle • Data storage and networking infrastructure • If it replaces several conventional scanners or breaks, no other scanners available

## Data Availability

Not applicable.
